# IT BOTHERS ME TO ASK FOR HELP: MODERATION OF SELF RELIANCE ON MASCULINE DEPRESSION AND SUICIDAL IDEATION IN OLDER MALES

**DOI:** 10.1093/geroni/igad104.2150

**Published:** 2023-12-21

**Authors:** Sabine Lohmar, Jeongwi An, Montgomery Owsiany, Erika Fenstermacher, Amy Fiske

**Affiliations:** West Virginia University, Morgantown, West Virginia, United States; West Virginia University, Morgantown, West Virginia, United States; West Virginia University, Morgantown, West Virginia, United States; West Virginia University, Morgantown, West Virginia, United States; West Virginia University, Morgantown, West Virginia, United States

## Abstract

Compared to females, males are less likely to seek help and less likely to be diagnosed with depression despite having depressive symptoms. Masculine depression, a ‘masked’ depression is associated with greater endorsement of masculine norms such as self-reliance. Self-reliance, or a preference to not seek help from others, is related to suicidal ideation in males. Although death by suicide remains the highest among adult males over the age of 75, research has yet to examine the role that self-reliance has on suicidal ideation in older males with masculine depression. This study examined the moderating effect of the self-reliance subscale of the Conformity to Masculine Norms Inventory (CMNI-30) on the relation between the Male Depression Risk Scale-22 (MDRS-22) and the Suicide Behaviors Questionnaires-Revised (SBQR). This study included 214 males aged 65 and older surveyed on Mechanical Turk. A multiple regression analysis indicated that self-reliance moderated the relation between the MDRS-22 and the SBQR (β =.220, t = 2.174, p=.031). Graphical representations of these data indicated that the relation between the MDRS-22 and SBQR was stronger in those with higher self-reliance. Thus, older males with masculine depression may be more likely to endorse suicidal ideation if they endorse self-reliance norms, or are less likely to seek help from others. This study illustrates the importance of self-reliance when understanding older males with elevated suicide risk. Future research should address how other masculine norms influence or explain the relation between masculine depression and suicidal ideation.

